# Interaction Network Construction and Functional Analysis of the Plasma Membrane H^+^-ATPase in *Bangia fuscopurpurea* (Rhodophyta)

**DOI:** 10.3390/ijms24087644

**Published:** 2023-04-21

**Authors:** Haiqin Yao, Wenjun Wang, Yuan Cao, Zhourui Liang, Pengyan Zhang

**Affiliations:** 1 Yellow Sea Fisheries Research Institute, Chinese Academy of Fishery Sciences, No. 106 Nanjing Road, Qingdao 266071, China; 2 Laboratory for Marine Fisheries Science and Food Production Processes, Laoshan Laboratory, Qingdao 266237, China

**Keywords:** hypo-salinity, interaction proteins, plasma membrane H^+^-ATPase, salt stress, seaweed, yeast two-hybrid

## Abstract

Salinity is a serious threat to most land plants. Although seaweeds adapt to salty environments, intertidal species experience wide fluctuations in external salinities, including hyper- and hypo-saline stress. *Bangia fuscopurpurea* is an economic intertidal seaweed with a strong tolerance to hypo-salinity. Until now, the salt stress tolerance mechanism has remained elusive. Our previous study showed that the expression of *B. fuscopurpurea* plasma membrane H^+^-ATPase (*BfPMHA*) genes were the most upregulated under hypo-salinity. In this study, we obtained the complete sequence of *BfPMHA*, traced the relative expression of this *BfPMHA* gene in *B. fuscopurpurea* under hypo-salinity, and analyzed the protein structure and properties based on the gene’s sequence. The result showed that the expression of *BfPMHA* in *B. fuscopurpurea* increased significantly with varying hypo-salinity treatments, and the higher the degree of low salinity stress, the higher the expression level. This BfPMHA had typical PMHA structures with a Cation-N domain, an E1-E2 ATPase domain, a Hydrolase domain, and seven transmembrane domains. In addition, through the membrane system yeast two-hybrid library, three candidate proteins interacting with BfPMHA during hypo-saline stress were screened, fructose–bisphosphate aldolase (BfFBA), glyceraldehyde 3-phosphate dehydrogenase (NADP^+^) (phosphorylating) (BfGAPDH), and manganese superoxide dismutase (BfMnSOD). The three candidates and *BfPMHA* genes were successfully transferred and overexpressed in a BY4741 yeast strain. All of them significantly enhanced the yeast tolerance to NaCl stress, verifying the function of BfPMHA in salt stress response. This is the first study to report the structure and topological features of PMHA in *B. fuscopurpurea* and its candidate interaction proteins in response to salt stress.

## 1. Introduction

Soil salinization is a global issue that threatens the productivity of plants, and estimates predict that 50% of global arable land will become salinized soil by 2050 due to the changes in the environment [1,2]. Soil salinization disrupts the biological uptake of nutrients and water, and thus produces physiological drought and ionic toxic effects on plants, disturbing necessary physiological functions required for plants’ growth and development [3]. The mechanism of plants’ response to salt stress is complicated and multifaceted, and still elusive. In marine organisms, almost all seaweeds that grow in intertidal zones suffer from salinity fluctuations caused by changes in tides and climates; thus, most of them possess tolerance to a wide range of salinity after a long period of evolution and adaptation to frequent changes in salinity [4]. For example, *Porphyra umbilicalis* can survive under salinities ranging from 7 to 52‰ [5]. Therefore, the large algae in intertidal zones were considered the optimal model organisms for investigating the mechanism of stress resistance of marine organisms and terrestrial plants [6]. 

The red algal genus *Bangia* is one of the oldest eukaryotes with sexual reproduction until now, and is found worldwide in both marine and freshwater habitats [7]. It has been reported that the only freshwater *Bangia* species, *Bangia atropurpurea*, and the most widely distributed marine species, *B. fuscopurpurea*, can adapt to each other’s habitat [8,9]. *B. fuscopurpurea* contains a large amount of proteins, free amino acids, polysaccharides, vitamins, and minerals, and it has been one of the most popular marine vegetables in China and commercially cultured in South China since the 1990s. Similar to laver, the intertidal mudflat culture mode is applied for aquaculture, where it frequently suffers a wide fluctuation of salinities, including hypo-salinity due to rainfall and land runoff [10]. Our previous studies indicated that the plasma membrane H^+^-ATPase (PMHA) may be an important enzyme that mediates the *Bangia* response to salinity fluctuation [11,12]. The gene of *B. fuscopurpurea* PMHA (*BfPMHA*) was the most upregulated among the numerous genes under hypo-salinity [13]. Furthermore, the inhibitor of BfPMHA decreased the tolerance of *B. fuscopurpurea* in response to hypo-salinity and *B. atropurpurea* to hyper-salinity [12]. 

PMHA belongs to the plasma membrane type (P-type) H^+^-ATPase superfamily, containing various numbers of transmembrane domains and many isoforms in various species [14,15]. The activity of PMHA is regulated at transcriptional, translational, and post-translational levels [16]. Plant PMHA includes N terminus, actuator domain, nucleotide-binding domain, phosphorylation domain, and C-terminal regulation domain. The C-terminal regulation domain contains two critical autoinhibitory regions and several functionally important phosphorylation sites, the status of which modulate the enzyme activity [17]. SOS_2_-like protein kinase 5 (PKS5) and 14-3-3 are the well-known proteins that interact with PMHA in plants. The evidence shows that the enzymes can stay in an active state through the phosphorylation of the autoinhibitory C-terminal, which promotes the binding of the 14-3-3 protein. However, the activity of PMHA is inhibited by PKS5 binding to the 14-3-3 protein in plants [18]. Based on the critical role of PMHA for plants to adapt to abiotic stress, this protein has attracted much attention. Accumulating studies have shown that environmental stresses such as salinity, alkalinity, nutrient deficiency, and low pH induce the expression of PMHA and enhance enzyme activities [19]. PMHA family genes or proteins have been identified and characterized in many plants such as cotton, grapes, tobacco, rice, rubber plant, and *Arabidopsis* [20]. Although the role of PMHA against stress has been investigated in some marine organisms [21,22,23], much less information is known about the *PMHA* genes and the mechanism of PMHA-induced salinity response in the *Bangia* species. 

To characterize the features of BfPMHA, we cloned the *BfPMHA* gene, traced this gene’s relative expression in *B. fuscopurpurea* under hypo-salinity stress, and analyzed the protein structure and properties based on the gene sequence. Moreover, to verify *BfPMHA* function in salt response, we transferred this gene into yeast and determined the tolerance ability and growth rate of the yeast under different NaCl concentrations. Finally, aiming to obtain more information about the mechanism through which BfPMHA mediates the *B. fuscopurpurea* response to salt stress, we screened the potential candidate proteins that interact with BfPMHA. The protein interaction experiments showed that fructose–bisphosphate aldolase (BfFBA), glyceraldehyde 3-phosphate dehydrogenase (NADP^+^) (phosphorylating) (BfGAPDH), and manganese superoxide dismutase (BfMnSOD) interacted with BfPMHA in vivo, thereby indicating that these three proteins may be involved in the *B. fuscopurpurea* response to salinity stress. Our study not only verified the function of BfPMHA but also preliminarily revealed the mechanism of how PMHA works in the *B. fuscopurpurea* response to hypo-salinity stress.

## 2. Results

### 2.1. Features of BfPMHA

**Figure 1:**
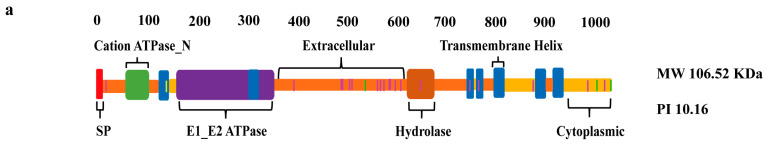

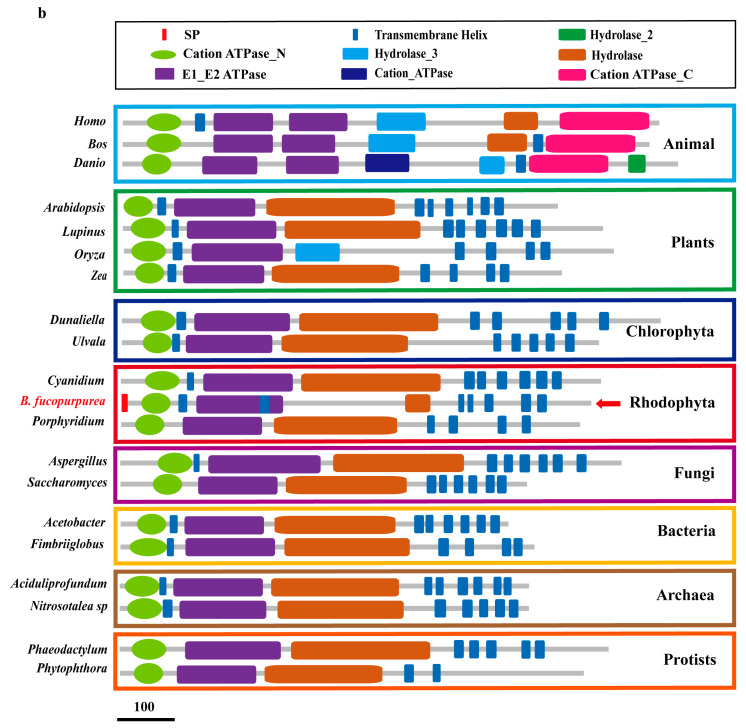
Schematic depiction of the structures of BfPMHA protein in *B. fuscopurpurea* (**a**) and PMHA in different organisms (**b**). SP: signal peptide.

The full sequence of *BfPMHA* was 3368 bp (GenBank: OQ363809) enriched with the GC content (up to 72%), including a 131 bp 5′-untranslated regions (UTR), a 108 bp 3′-UTR, and a 3129 bp coding sequence (CDS), and encoding a 1042-amino-acid polypeptide (predicted MW 106.51 kDa, PI 10.16) (Figure 1a). The structural prediction of BfPMHA indicated that this protein has a signal peptide (SP) (aa 1–17). The BfPMHA contained three amidation sites (covered by green arrows), nineteen protein kinase C phosphorylation sites (covered by pink arrows), one N-glycosylation site (covered by yellow arrows) (Figure 1a), seven predicted transmembrane domains (TMDs) (aa 128–146, 307–330, 342–363, 369–391, 804–826, 888–909, and 921–945), a cation ATPase_N domain (aa 49–114), an E1–E2 ATPase domain (aa 160–371), and a hydrolase domain (aa 627–695) (Figure 1a). The hydrolase domain (covered by brown arrows) included a phosphatase domain and a kinase domain, which functioned in dephosphorylating and phosphorylating the BfPMHA, respectively. The structural features of BfPMHA and BfPMHA-like proteins from different organisms were also annotated. These proteins were very conservative, mainly features with Cation ATPase_N, E1_E2 ATPase and TMD domains (Figure 1b). These proteins can be roughly divided into two groups based on the number of E1_E2 ATPase domains, with animal PMHAs making up one group and others making up the other group. Besides the BfPMHA and animal PMHAs, PMHAs in other organisms possess a relative long hydrolase domain that contains a phosphatase domain and a kinase domain and functions in dephosphorylating and phosphorylating the protein. 

**Figure 2:**
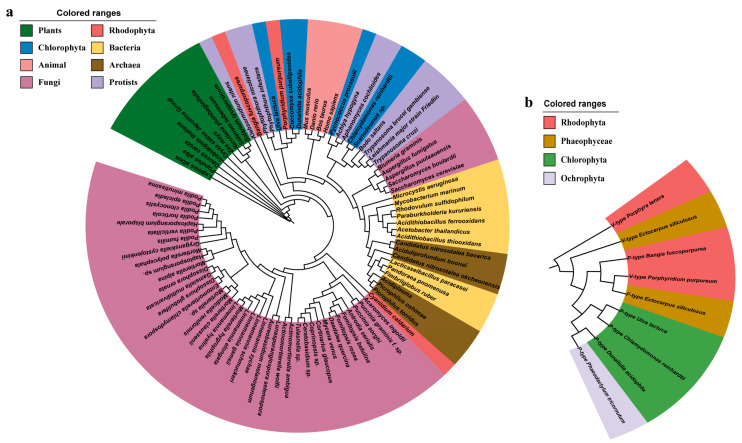
Phylogenetics of subsets of PMHA and H^+^-ATPase amino acids. (**a**) Phylogenetic analysis of PMHA protein in different organisms. (**b**) Phylogenetic analysis of H^+^-ATPase in algae.

A phylogenetic tree base on the PMHA sequences is shown in Figure 2a. The tree was characterized by a crosslink among organisms; for example, PMHAs from Rhodophyta were categorized into three branches, those close to protists, chlorophyte, and fungi, respectively. The analysis result showed that BfPMHA shared a high similarity with orthologous proteins from these branches. The data on P-type H^+^-ATPase in algae shared online are very limited, and particularly rare on red algae. Here, we collected few H^+^-ATPases, belonging to different H^+^-ATPase types based on the cellular localization. Via the database of NCBI, we analyzed the protein sequences of these three types of H^+^-ATPase in algae species. With the exception of the BfPMHA, the H^+^-ATPase in other red algae is mainly the vacuolar type (V-type), in brown algae it is the mitochondrial/chloroplast type (F-type) or V-type, and in green algae it is the P-type (Figure 2b). BfPMHA was close to the V-type H^+^-ATPase in *Porphyridium purpureum* and the F-type H^+^-ATPase in *Ectocarpus siliculosus*. 

### 2.2. Expression Pattern of the BfPMHA Gene under Hypo-Salinity

**Figure 3:**
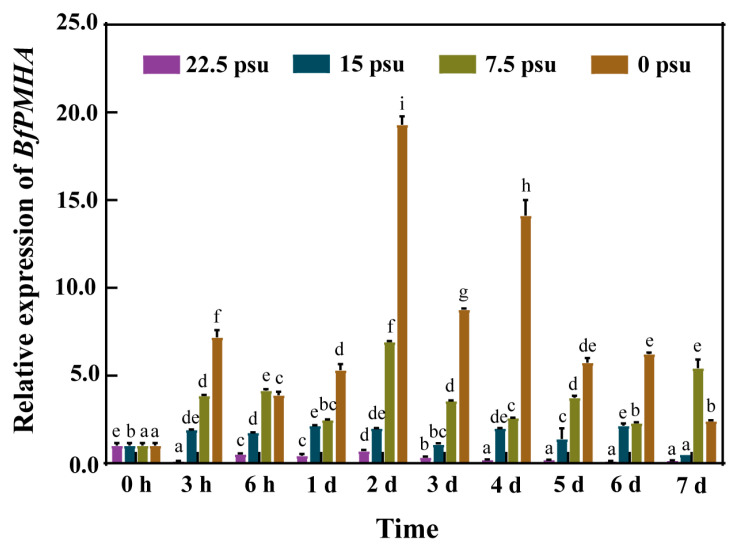
The relative expression of the *BfPMHA* gene under hypo-saline stress with *ef1γ* gene as reference. The values are represented as mean ± SD of three replicates. Error bars indicate standard error of the mean. Different letters indicate significant differences (*p*-value < 0.05).

To investigate whether *BfPMHA* was involved in salinity responses in *B. fuscopurpurea*, the expression of this gene in gametophytic thalli was investigated under different salinities using qRT-PCR. The expression of the *BfPMHA* gene was significantly higher than in the 30 psu group during the 7 days of treatment. Generally, the higher the degree of low salinity stress, the higher the expression level of *BfPMHA*. *BfPMHA* exhibited the highest expression at 48 h in the 0 psu group (Figure 3), a value 20 times higher than in 30 psu group. This result suggested that the *BfPMHA* functioned in the *B. fuscopurpurea* response to hypo-salinity stress.

### 2.3. Screening of Putative Interaction Proteins of BfPMHA

**Figure 4:**
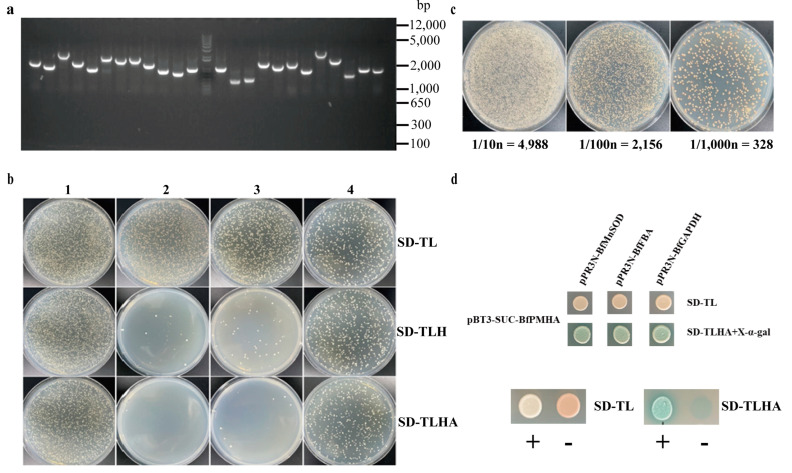
Screening of yeast two-hybrid system. (**a**) PCR amplification of inserted fragments in secondary library. (**b**) The result of *BfPMHA* auto-activation and function verification. (**c**) The transformation efficiency of the library screened by *BfPMHA*. (**d**) Yeast two-hybrid assay of interesting proteins in *B. fuscopurpurea* hypo-saline stress response, manganese superoxide dismutase (BfMnSOD), fructose-bisphosphate aldolase (BfFBA), glyceraldehyde 3-phosphate dehydrogenase (BfGAPDH, NADP^+^, phosphorylating). 1: positive control, 2: negative control, 3: no activation, 4: functional. SD-TL: SD/-Leu/-Trp, SD-TLH: SD/-Leu/-Trp/-His, SD-TLHA: SD/-Leu/-Trp/-His/-Ade (+X-α-gal), +: positive control, -: negative control.

To gain an insight into the function and regulation of BfPMHA and potentially identify new signaling pathways involved in *B. fuscopurpurea* salt tolerance, the putative interaction protein of BfPMHA was investigated by H2Y. The size of the cDNA library was 1.15 × 10^7^ CFU, the average size of the inserted fragments was larger than 1.2 kb (Figure 4a), and the positive rate of the *B. fuscopurpurea* cDNA library was 100%. The result of the auto-activation assay showed that pPR3N and pBT3-SUC-BfPMHA co-transgenic yeast cells were able to grow well on the SD-TL, whereas yeast cells with pPR3N/pBT3-SUC-BfPMHA could not grow on the selected plates (Figure 4b). These results indicated that the bait fusion protein was well expressed without self-activation. Using the bait pBT3-SUC-BfPMHA to screen the *B. fuscopurpurea* cDNA library produced a transformation efficiency of 3.16 × 10^5^/ug (Figure 4c), indicating that the library can cover the *B. fuscopurpurea* cDNA library well. 

Finally, 31 positive clones were obtained and confirmed through re-streaking on selected plates, with the results partially exhibited in Figure 4d. A subsequent bioinformatic analysis was performed to obtain the sequence information of these positive clones. The results showed that 13 reads encoded 12 different proteins (Table S3), including MnSOD, FBA, GAPDH. These three proteins have been reported to play roles in the plant response to stress. Here, they interacted with BfPMHA and may have been involved in the *B. fuscopurpurea* response to hypo-salinity.

### 2.4. Characterization of BfMnSOD, BfFBA, and BfGAPDH

**Figure 5:**
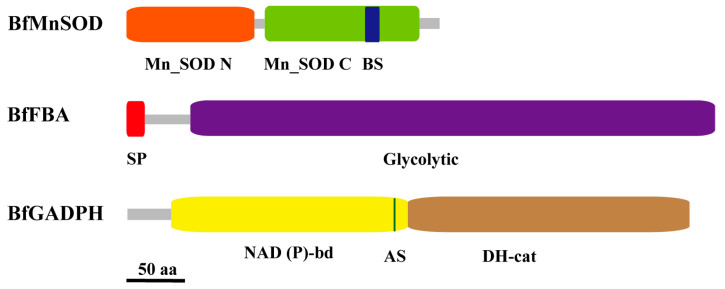
Schematic depiction of the structures of three proteins, BfMnSOD, BfFBA, and BfGAPDH. Scale bar is 50 amino acids. BS, bind site. SP, signal peptide. AS, active site. DH, dehydrogenase.

The full-length cDNA of *BfMnSOD*, *BfFBA*, and *BfGAPDH* was isolated from the cDNA library and sequenced. The results indicated that the CDS of *BfMnSOD* was 675 bp, encoding a 225-amino-acid protein that contains N and C domains of Mn-SOD; the CDS of *BfFBA* was 1242 bp, encoding a 414-amino-acid protein that contains a glycolytic domain; the *BfGAPDH* was 1134 bp, encoding a 378-amino-acid protein that contains a NAD(P)-bd domain and a DH-cat domain (Figure 5).

### 2.5. Analysis of Interaction between BfPMHA and the Three Interaction Proteins

**Figure 6:**
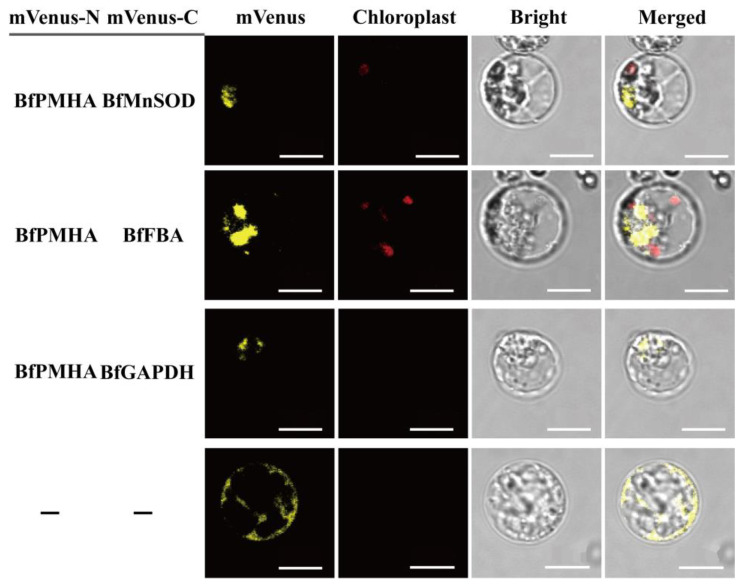
BfPMHA separately interacts with BfMnSOD, BfFBA, and BfGAPDH in rice protoplasts by BIFC assays. mVenus, yellow fluorescence. Chloroplast, red fluorescence. Scale bar, 10 μm. The negative control of BiFC is shown in Supplementary Figure S1.

We selected a membrane-based yeast two-hybrid system for yeast two-hybrid screening because of the localization of BfPMHA in the cell membrane. These interactions of BfPMHA with BfMnSOD, BfFBA, and BfGAPDH at the cell membrane were separately confirmed via bimolecular fluorescence complementation in rice protoplasts (Figure 6). These results indicate that BfPMHA can directly interact with BfMnSOD, BfFBA, and BfGAPDH in the cell membrane and can form homodimers and/or heterodimers. This is in accordance with our predictions.

### 2.6. Transgenic Yeast Tolerance to NaCl Stress

**Figure 7:**
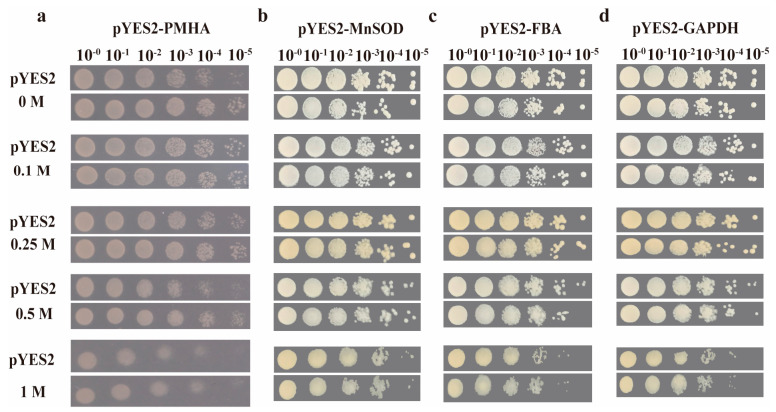
The salt tolerance ability of transgenic yeast. The salt tolerance ability of pYES2 strain with overexpressed genes of *BfPMHA* (**a**), *BfMnSOD* (**b**), *BfFBA* (**c**) and *BfGAPDH* (**d**) under the different NaCl concentrations, with the empty pYES2 strain as control. The concentrations of NaCl include 0 M, 0.1 M, 0.25 M, 0.5 M, and 1 M, each NaCl concentration with 5 times gradient dilution. M, mol/L.

In order to better evaluate the function of BfPMHA and the three interacting proteins BfFBA, BfGAPDH, and BfMnSOD in salt stress, respectively, we cultured transgenic yeast at different NaCl concentrations to observe the growth condition of this yeast, which was transformed with these genes. The result showed no difference in growth between the *BfPMHA* of the transgenic and control (transformed empty vector pYES2) yeast under normal culture conditions (Figure 7a). Compared with the control yeast, pYES2-*BfPMHA*-overexpressing yeast a showed faster growth rate, but both pYES2-*BfPMHA*-overexpressing yeast and the control yeast could not survive when the NaCl concentration was higher than 2 M. The results of the three *BfPMHA* interaction genes were the same as the effect of the overexpressing *BfPMHA* in yeast. The growth rate of the transgenic yeast with *BfMnSOD* (Figure 7b), *BfFBA* (Figure 7c), and *BfGAPDH* (Figure 7d) was higher than that of the yeast strain that merely contained a pYES2 vector under NaCl stress.

**Figure 8:**
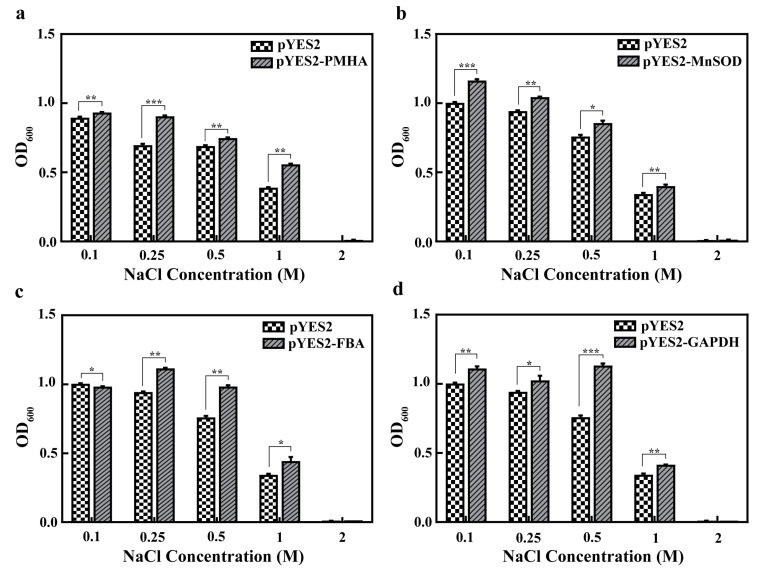
Expression pattern analysis of *BfPMHA* (**a**), *BfMnSOD* (**b**), *BfFBA* (**c**), and *BfGAPDH* (**d**) in yeast under various NaCl concentrations. M: mol L^−1^. The values are represented as mean ± SD of three replicates. Error bars indicate standard error of the mean, *, **, and *** indicate significant differences (*p*-value < 0.05, *p*-value < 0.01 and *p*-value < 0.001).

To quantify the growth rate between four genes overexpressed in yeast and control yeast, the cell densities (OD_600_) of the yeast were measured under different NaCl concentrations. The result showed that there was no difference between the cell densities under normal conditions. The cell densities of *BfPMHA* transgenic yeast were significantly higher (*p* < 0.05) than those of the control after NaCl treatment, particular in the concentration of NaCl higher than 0.25 M (Figure 8a). The cell densities of both *BfPMHA* transgenic yeast and the control yeast showed a decline with the increasing concentration of NaCl, and the yeast growth was inhibited by more than 2 M NaCl. The transgenesis of *BfMnSOD*, *BfFBA*, and *BfGAPDH* all significantly (*p* < 0.05) increased the yeast growth rate with 0.1–1 M NaCl treatment (Figure 8b–d). The results indicate that *BfPMHA*, *BfMnSOD*, *BfFBA*, and *BfGAPDH* increased the yeast tolerance to NaCl.

## 3. Discussion

The complete H^+^-ATPase gene of *B. fuscopurpurea* obtained in this study was a plasma membrane H^+^-ATPase. Additionally, this was the first PMHA isolated and identified in red algae. The BfPMHA contained very high GC content, up to 72%. A high GC content in the protein coding regions seems to be a common property of Bangiales. The GC content of the *Porphyra umbilicalis* CDS ranges from 72.9% to 94% based on genome analysis [6]. The high GC content increased the difficulty of the works carried out on the BfPMHA gene cloning. Analyzing the BfPMHA protein and the PMHA of other taxons, we found that these proteins are characterized with TMDs, Cation ATPase_N, E1-E2 ATPase, and Hydrolase, which includes a phosphatase domain and a kinase domain, which function in dephosphorylating and phosphorylating, respectively. The TMDs are one of typical features of the PMHA (Figure 1), which belong to the P3A subfamily [24]. Plant PMHA contains about 10 TMDs [25]. Based on the structure analysis of the BfPMHA protein, these results indicate that it belongs to a plasma membrane protein. TMDs are vital for the ability of proteins to perform diverse functions, such as cellular recognition, molecular receptor activity, signal transduction, and enzymatic activity [26]. The evidence shows that transmembrane helices are a tightly packed conformationally sensitive domain, which can transmit a conformational change to the active site of the enzyme in yeast [27]. However, the C-terminal autoinhibitory domain was not observed in the red algae *B. fuscopurpurea*. The evolution of the core structure of the plant PMHA showed that the C-terminally conserved phosphorylation site does not exist in red algae [28]. In addition, a homologous PMHA sequence corresponding to the autoinhibitory domain neither found in the red algae *Cyanidium caldarium* was probably involved in strong acid-tolerance of *Cyanidium caldarium* [29]. The BfPMHA of *B. fuscopurpurea* also seems to contain many low-complexity domains that are intrinsically disordered and not amenable to conventional structure–function analysis [30], which probably leads to the low genetic homology to the other PMHA proteins. PMHA transformation is regulated by phosphorylase treatment, which causes coupling bio-reactions of ATP hydrolysis and proton pumping [31]. Phosphorylation is one of the most important modifications after protein translation. In general, a protein is activated with phosphorylation and inactivated with dephosphorylation or activated with dephosphorylation and inactivated with phosphorylation. Many studies have been investigated in higher plants and confirmed that the PMHA activity was closely related to the level of phosphorylation [32,33,34]. In wheat, more than 40 phosphorylation functional sites of PMHA protein have been identified [35]. In this study, a total number of 77 phosphorylation sites were predicted to be present in the BfPMHA protein. Overall, the structural characteristics of BfPMHA in red algae probably contribute to their well-adaptation to extreme environments such as desiccation and salinity. However, further investigation is warranted. Moreover, this protein has potential for use in plant transformation to improve plant tolerance to salt stress via the application of gene-editing tools, such as the CRISPR/Cas9 system. Editing the promoter region of PMHA genes would allow the breeding of plants with higher yields [14].

Phylogenetic analysis of the PMHA protein sequences from diverse organisms has shown that the PMHA is diverse within the same organism. Falhof et al. [28] demonstrated a relatively large sequence variation in the catalytic core within and between fungi, red algae, and green algae, which may indirectly support the diverse sequence in red algae. However, the tree also showed that BfPMHA was distinct from the higher plants and fungi. The complex structure and diverse distribution of PMHAs have been suggested to have high bioactivity, play crucial roles in bioregulation, and help various organisms adapt to varying environmental changes [36]. The known H^+^-ATPases are classified into three types: P-type, V-type, and F-type. They are important ATP-driven proton pumps that create membrane potential and provide a proton motive force for secondary active transport [37]. However, the database of P-type H^+^-ATPases genes are relatively smaller for algae, especially red algae, which is probably because less research is performed on algae. For example, the proton-pumping of plasma membrane preparation vanadate-sensitive was demonstrated in only few algal species, and ATPase protein purification was demonstrated only in *Dunaliella acidophila* [21]. Another reason that should be considered is that seaweeds, especially red seaweeds, are quite different from higher plants due to the high repeat component and GC content of Bangiales [13,38]; thus, there are very few reports about the gene cloning and function verification testing. Through analyzing the reported H^+^-ATPase proteins in algae, we found that the present reported BfPMHA was the first PMHA reported in the red algae *Bangia*, and the genetic distance of this BfPMHA was close to the V-type H^+^-ATPase of *Porphyridium purpureum* and the F-type H^+^-ATPase of *Ectocarpus siliculosus* (Figure 2b), suggesting that the three types H^+^-ATPase share similarity in structure and function. A previous study showed a relative short distance between red algae H^+^-ATPase and AHA10 (H^+^-ATPase 10 of *Arabidopsis*). The AHA10-related proteins are distinct from PMHA in that they target the tonoplast and have a vacuolar membrane targeting signal [39,40]. Although the F-type and V-type H^+^-ATPase show distinct functions in plants, the general structures of the F-type and V-type are remarkably similar [41]. Moreover, the PMHAs from the different lineages were clustered together (Figure 2a), indicating that the PMHAs were highly conservative [14], but there were certain differences among different organisms. Overall, due to their similar structures and functions, P-type, V-type, and F-type H^+^-ATPase are highly conserved across species and share a significant degree of sequence similarity. This conservation suggests that these enzymes probably evolved to perform similar functions and play crucial roles in maintaining cellular energy and ion homeostasis in cytoplasm or in organelles [42].

Many studies have reported that PMHAs play roles in the regulation of many physiological processes fundamental for plant growth and development [43], metal absorption [44], stomatal aperture and gas exchange [45], pH homeostasis in cytosol, cell expansion [46], cellular expansion [47], etc. The crucial role of PMHA in the resistance of plants to diverse abiotic stresses, such as cold [48], drought [49], and salt stress [50,51], has also been well documented. The previous data from our team indicate that the expressed sequence tags (ESTs) of BfPMHA were the highest upregulated among all the identified ESTs in *B. fuscopurpurea* after 6 h of 90% freshwater treatment [13]. The PMHA catalyzes the hydrolyzing of ATP and generates energy, which pumps H^+^ out of the plasma membrane and thus creates and maintains a negative membrane potential and a transmembrane proton gradient [52]. The PMHA inhibitor sodium vanadate (Na_3_VO_4_), extensively used in studying the activity and function of this enzyme [53], inhibited the speed of the H^+^ transition across the plasma membrane of *B. fuscopurpurea* and *B. atropurpurea* under hypo- or hyper-salinity stresses; however, the Na^+^ efflux was significantly increased [54]. This result was contrary to the assumption that Na_3_VO_4_ inhibits the activity of BfPMHA. This probably because Na_3_VO_4_, as an inhibitor of PMHA, is also a sodium salt. The *B. fuscopurpurea* was under relatively higher Na^+^ stress than that in the control group after adding Na_3_VO_4_, which might stimulate other regulatory functions, such as Na^+^/H^+^ antiporter [55] effects on *B. fuscopurpurea* to increase the Na^+^ efflux. Moreover, as the concentration of Na_3_VO_4_ and the treatment period increased, the inhibitory effects on the Fv/Fm and Pn of *B. fuscopurpurea* and *B. atropurpurea* during salinity stress were aggravated [12]. Salinity stress results in the accumulation of excess ions, such as calcium (Ca^2+^), magnesium (Mg^2+^), sodium (Na^+^), sulfates (SO_4_^2−^), and chlorides (Cl^−^). The toxic effects of Na^+^ and Cl^−^ are major contributory factors for ionic imbalance in plants [45]. Establishing a new ionic equilibrium is an important way for plants to tolerate salt. The cooperation of transporters of Na^+^/H^+^ and PMHA on plasma membrane excretes Na^+^ from the cell and hence improves the tolerance to salt stress in plants. In this study, the relative expression of BfPMHA was significantly upregulated with 3 h of hyposaline treatment, and it increased with the increase in hyposaline stress (Figure 4). The transgenesis of this gene increased the transgenic yeast’s tolerance to NaCl and the cell growth under different NaCl concentrations (<2 M). Numerous of studies have reported that the PMHA genes play an important role during salt stress response in plants [56,57]. However, to the best of our knowledge, there are no reported studies on the salt stress resistance of the PMHA genes in red algae. Here, we not only cloned, characterized, and analyzed the BfPMHA in the red alga *B. fuscopurpurea* during hypo-saline stress, but also successfully expressed this gene in yeast. In conclusion, these results showed that BfPMHA mediated *B. fuscopurpurea* in response to salinity stress.

BfMnSOD, BfFBA, and BfGAPDH were screened out and verified to be the potential interaction proteins of BfPMHA. They were able to improve the growth rate of the transgenic yeast under different NaCl concentrations (Figure 7 and Figure 8). All these results indicated that BfPMHA, BfFBA, BfGAPDH, and BfMnSOD are potential candidate genes for responding to salt stress, which provided a new insight into the mechanism of *B. fuscopurpurea* acclimating to salinity fluctuation. Interestingly, a common feature of these proteins is that they are directly or indirectly related to the antioxidant response to abiotic stress. The MnSOD catalyzes the disproportionation of superoxide into H_2_O_2_ and O_2_^•−^, which has long been regarded as a bystander anti-oxidase to prevent transient superoxidase from exploding [58]. However, recent reports have demonstrated that MnSOD functions as a thermoreceptor to regulate O_2_^•−^/H_2_O_2_ homeostasis and fluxes [59]. McAdam et al. [60,61] found that the gating ratio of this enzyme’s activity was only regulated by the temperature. Zhang et al. [59] found a “set point” temperature, at which all reactions of this enzyme were switched to fast cycles. Our previous study revealed that the SOD activity was rapidly induced under saline stress and there was no significant accumulation of O_2_^•−^ in *B. fuscopurpurea* and *B. atropurpurea*, indicating that SOD plays an essential protective role in the *B. fuscopurpurea* response to hypo-salinity and in the *B. atropurpurea* response to hyper-salinity [11]. Nounjan et al. [62] found that salt stress induced the expression of MnSOD, agreeing with Kaminaka et al. [63] and Sairam et al. [64]; they indicated that the MnSOD gene or the activity of MnSOD were strongly enhanced by salinity. In short, these clues show that MnSOD is involved in the plant salt stress response. The GAPDH coverts glyceraldehyde-3-phosphate to D-glycerate 1,3-bisphospate in the presence of nicotinamide adenine dinucleotide (NAD^+^) and inorganic phosphate and mediates the formation of NADP and adenosine triphosphate (ATP) in the cytoplasm. GAPDH has been considered a classical glycolytic protein over the past few decades. Recently, accumulating evidence has shown that post-translational modifications of mammalian GAPDH function in many cellular processes, such as DNA repair, tRNA export, membrane fusion and transport, cytoskeletal dynamics, cell death, and probably the stress response, apart from glycolysis [65]. Although the role of GAPDH in higher plants has not been widely explored, it has already been proven to modulate plants’ immunity [66,67]. In *Nicotiana benthanmiana*, GAPDH acted as a suppressor of autophagy, and its function could be carried out by its interaction with autophagy-related genes or proteins [68]. The overexpression, knockout, or silence of GAPDH increased ROS levels and the autophagy ability in *Arabidopsis* and tobacco [69]. Wawer et al. [70] presented that GAPDH as a cellular partner is present in the immunocomplex with NtOSAK (*Nicotiana tabacum* osmotic stress-activated protein kinase) in salt-treated *N. tabacum* cells. In addition, Nakajima et al. [71] announced that aggregate formation is a well-known feature of GAPDH. Altogether, GAPDH can play a role in the plant salt stress response. The FBA is a vital enzyme that participates in plants’ carbohydrate metabolism, including glycolysis, gluconeogenesis, and the Calvin cycle [72]. This gene is expressed in various tissues of plants, suggesting that it may function at different stages of growth and development [73]. Accumulating evidence has revealed different expression patterns of FBA genes in response to varying stresses, such as hyper-saline stress [74], abnormal temperature [75], and drought [76]. The roles of FBA in response to abiotic stresses have been well investigated in higher plants [75,77,78,79]. For example, the activity of FBA was upregulated in wheat seedlings under salt stress, which improved the seedlings’ adaption to the stress [80]. The role of FBA playing under salt stress is probably because it affects the accumulation of malonaldehyde (MDA), which leads to DNA and biological membrane damage, and therefore protects the cell membrane under salt stress [72,81]. Previous reports showed that during oxidative stress, the accumulation of MDA content decreased in *SiFBA5*-overexpression transgenic lines [82]. During tomato chilling stress, the MDA content was significantly lower in *SlFBA4*-overexpressing seedlings than that in RNAi seedlings [72]. Wang et al. [11] revealed that the antioxidant enzyme activities in hypo-salinity-treated *B. fuscopurpurea* were very different from those in *P. haitanensis* and *P. yeoensis*, indicating the unique function of *B. fuscopurpurea* acclimating to hypo-salinity. Furthermore, the regulation of PMHA mainly occurs after post-translation. Some known post-translational regulations such as PKS5 [83,84], peptide-containing sulfated tyrosine 1 receptor (PSY1R), FERONIA (FER), abscisic acid insensitive 1 (ABI1), and 14-3-3 [85] proteins have been well identified in higher plants. PMHA, as a membrane protein that interacts with proteins inside the cell, such as 14-3-3, PKS5, etc., due to their N- and C-terminal domains, was exposed on the cytosolic side of the membrane [28]. However, these documented regulators of PMHA in higher plants were not identified in *B. fuscopurpurea* under hypo-salinity stress. Previous evidence showed that FBA may function in the crosstalk of salt-signaling pathways, and several FBA proteins from tomato were grouped together with NpAldp1 and NpAldP2, which are involved in salt stress [74]. FBA overproduction increased tobacco salt stress tolerance via an increase in proline [73]. Moreover, *fba6* mutants displayed a higher germination rate than the WT plants when they were germinated in medium containing 125 mM NaCl. Taken together, these findings illustrate that the FBA gene family indeed plays important roles not only in development but also in salt response. 

However, the physiological role of these protein interactions during salt stress is not well understood in *B. fuscopurpurea*. They are probably involved in regulating and protecting the cell membrane from oxidative damage to stabilize the activity of BfPMHA in *B. fuscopurpurea* during hypo-salinity stress. Further investigation on the physical functions of these potential interaction proteins would help dissect the regulatory network of BfPMHA under hypo-salinity stress. The interaction between proteins can play specific biological functions, such as maintaining the structure of proteins, regulating metabolic processes, transmitting cellular signals, and so on [86,87]. All these proteins were reported to play roles during the salt stress. Although there is no evidence to show these proteins’ interaction in the plant response to salt stress, we did not conduct a further investigation in this study. Nevertheless, the transgenic yeast in this study showed that these proteins were activated, and they increased cells’ tolerance to hyper-salinity stress. In addition, as there are few studies on how BfPMHA-mediated *B. fuscopurpurea* responds to hypo-salinity stress at the molecular level, the BfPMHA function and the interaction mechanism between BfPMHA and its interaction proteins are worth further investigation in the future.

## 4. Materials and Methods

### 4.1. Materials and Growth Conditions

The gametophytic thalli of *B. fuscopurpurea* were collected from a farm in Putian China (119.47° E, 25.22° N) and stored at −20 °C. Five grams of thalli was cultured in 100% seawater, 75% seawater, 50% seawater, and 25% seawater diluted with sterilized freshwater (i.e., salinity of ~30 psu, 22.5 psu, 15 psu, 7.5 psu), and 100% sterilized freshwater (i.e., salinity of ~0 psu), respectively. Each treatment was performed in triplicate. Samples were collected after 3 h, 6 h, 1 d, 2 d, 3 d, 4 d, 5 d, 6 d, and 7 d of treatment, respectively, for qRT-PCR tests. After collecting, fresh thalli were immediately frozen in liquid nitrogen. 

### 4.2. Isolation and Sequence Analysis of BfPMHA

The total RNA was extracted with RNeasy Plant Mini Kit and RNase-Free DNase Set (Qiagen, Duesseldorf, Germany) as per the manufacturer’s instructions, and used to generate a 5′/3′-RACE read with the GeneRacerTM Kit (Invitrogen, Carlsbad, CA, USA) as per the manufacturer’s instructions. The primers used are listed in Table S1.

### 4.3. Prediction of BfPMHA Sequence Features and Protein Structure

The full-length CDS was predicted by ORF Finder (http://www.ncbi.nlm.nih.gov/orffinder/, accessed on 31 March 2021), and the basic physical and chemical parameters of the putative protein were calculated by ProtParam (http://web.expasy.org/protparam/, accessed on 13 April 2021). PredictProtein (https://www.predictprotein.org/, accessed on 14 April 2021) combined with PFAM (http://pfam.sanger.ac.uk, accessed on 14 April 2021) and InterProScan (https://www.ebi.ac.uk/interpro/search/sequence/, accessed on 14 April 2021) was used for motif and domain analyses. Signal peptide (SP) sequences were analyzed by SignalP 6.0 (https://services.healthtech.dtu.dk/service.php?SignalP-5.0, accessed 15 April 2021) and ChloroP1.1 (http://www.cbs.dtu.dk/services/ChloroP/, accessed on 14 April 2021), respectively. The NetPhos 3.1 server (http://www.cbs.dtu.dk/services.php?NetPhos-3.1, accessed on 19 April 2021) was used to predict sites for serine, threonine, and tyrosine phosphorylation in this protein. The protein subcellular localization was predicted by TargetP (http://www.cbs.dtu.dk/services/TargetP, accessed on 18 April 2021). The online CCTOP (http://cctop.enzim.ttk.mta.hu/?_=/jobs/submit, accessed on d 18 April 2021) tool was used to identify the BfPMHA transmembrane topology. Phylogenetic analysis by the neighbor-joining method was performed using MEGA 7.0, and the annotation was conducted with iTOL software (http://itol.embl.de/itol.cgi, accessed on 19 April 2021). In this paper, all the H^+^-ATPase gene sequences of other organisms were retrieved from NCBI, and the details of these sequences are provided in Table S2 attached. 

### 4.4. RNA Isolation and qRT-PCR

The total RNA was extracted with RNeasy Plant Mini Kit and RNase-Free DNase Set (Qiagen, Duesseldorf, Germany). Approximately 100 ng of total RNA was reverse transcribed, and the first strand of cDNA was obtained by reverse transcription by using Superscript Ⅲ reverse transcriptase kit (Invitrogen, Carlsbad, CA, USA) as per the manufacturer’s instructions. qRT-PCR experiments were performed using Power SYBR^®^ Green PCR Master Mix (Applied Biosystems, CA, USA) in the Bole CFX 384 Multi-channel Fluorescence Quantitative PCR (Bio-Rad, Hercules, CA, USA). Each sample was performed in triplicate, using the 2^−ΔΔCT^ method to calculate the relative expression [88]. The *B. fuscopurpurea ef1γ* gene was used as an internal reference. The primers used are listed in Table S1.

### 4.5. Protein Interaction Analysis

To obtain more relative genes that respond to hypo-salinity stress, a cDNA library was constructed. About 5 g fresh gametophytic thalli from *B. fuscopurpurea* was collected after 48 h of 0 psu treatment. Total RNA was extracted using TRIzol (Invitrogen, Carlsbad, CA, USA) and isolated using Oligotex mRNA Mini kit (Qiagen, Duesseldorf, Germany) according to the manufacturer’s instructions. About 4.5 μg of RNA was used for synthesizing the first- and the double-stranded cDNA according to the protocol as described above. The cDNA fragments were ligated into pPR3-N fusion vector as prey proteins. The ligation mixture was transferred into the competent cells of *Escherichia coli* DH5α by electroporation (Bio-Rad, CA, USA). The titer of the primary cDNA library was calculated according to the clone numbers on the plates. PCR was used to confirm the size of the inserted fragments in the library.

The membrane system yeast two-hybrid (Y2H) library was constructed to screen the library. The ligation vector was pPR3-N, and the *BfPMHA* was constructed on the pBT3-SUC vector and used to screen the library. The plasmids pNubG-Fe65 and pTSU2-APP were co-transformed as the positive control, taking the pPR3-N and pTSU2-APP as the negative control, and the pPR3-N and pBT3-SUC-*BfPMHA* as the self-activation control. The plates were subject to β-galactosidase activity assay using X-α-Gal staining. The β-galactosidase activity was determined using the HTX High-throughput β-galactosidase Kit (Dual membrane systems, Biotech, Switzerland). The yeast culture temperature was 30 °C, and the 3-aminotriazole (3-AT) screening concentration was 5 mM.

The bimolecular fluorescence complementation (BiFC) assay was performed in rice protoplasts. Rice protoplast isolation and transformation were performed according to the previously described method [89,90]. The *BfPMHA* gene was cloned into the 1300s-mCherry-N vector, and its candidate interaction protein genes *BfMnSOD*, *BfFBA*, and *BfGAPDH* were cloned into the 1300s-mCherry-C vector [76]. Rice protoplasts were isolated from 12-day-old seedlings of ZH11 (*Oryza sativa* spp. *japonica*) according to Hu et al. [91].

### 4.6. NaCl Stress Tolerance Assay in Yeast

Yeast expression systems were performed to analyze the potential role of the BfPMHA, BfMnSOD, BfFBA, and BfGAPDH in salt stress tolerance. All amplified fragments were sub-cloned into the pYES2 vector (AngYuBio, Shanghai, China), which contained the URA3 selection markers driven by a GAL1 promoter, and the constructed vectors were introduced into yeast BY4741 (AngYuBio, Shanghai, China) [92]. All primers used in this experiment are listed in Table S1. The yeast transformants were cultured in liquid SC-Uracil medium that contained 2% (*w*/*v*) galactose for overnight at 30 °C. To promote the expression of these genes, the cultures were adjusted to an OD_600_ of 0.4 with liquid SC-Uracil medium that contained different concentrations of NaCl (0 M, 0.1 M, 0.25 M, 0.5 M, 1 M, 2 M, 3 M, 4 M, and 5 M) and were grown at 30 °C with shaking. The cell density (OD_600_) was recalculated after 24 h, then adjusted to an OD_600_ of 0.4 and deposited on the solid SC-Uracil medium that contained 2% (*w*/*v*) galactose. 

## 5. Conclusions

The function of PMHA in mediating hypo-salinity tolerance in the red algae *B. fuscopurpurea* is first reported in this study. PMHA plays an essential role in regulating hypo-salinity tolerance by upregulating gene expression. The *BfPMHA* increased significantly with varying hypo-salinity treatments, and the higher the degree of low salinity stress, the higher the expression level. Furthermore, this study presented the first report on constructing the interaction network and analyzing the function of the plasma membrane H^+^-ATPase of red algae in vitro. Three screened BfPMHA interaction proteins, FBA, GAPDH, and MnSOD, have been reported to play roles in the plant stress response, and they interacted with BfPMHA in vivo and increased the ability of yeast to respond to salt stress, respectively. Therefore, we suggest that *BfPMHA* plays active roles against hypo-salinity in *B. fuscopurpurea*. This protein has the potential to be used in plant transformation to improve the plant tolerance to salt stress via the application of gene-editing tools to improve the yield production of plants.

## Data Availability

The data presented in this study are available in the article and Supplementary Materials.

## Supplementary Materials

The supporting information can be downloaded at: https://www.mdpi.com/article/10.3390/ijms24087644/s1.
