# Supplementary material for: Interaction Network Construction and Functional Analysis of the Plasma Membrane H+-ATPase in Bangia fuscopurpurea (Rhodophyta)
Source: Int J Mol Sci. 2023 Apr 21;24(8):7644. doi: 10.3390/ijms24087644 (PMC10142769; doi:10.3390/ijms24087644)
Supplement: Supplementary file 1 [file ijms-24-07644-s001.zip › ijms-2248020-supplementary.pdf]

Table S1 Primer sequences for molecular experiments. BfPMHA: plasma membrane H^+-ATPase of *B. fuscopurpurea*; Eflγ: elongation factor 1-gamma; BfFBA: fructose-bisphosphate aldolase; BfGAPDH: glyceraldehyde 3-phosphate dehydrogenase ($NADP^+$) (phosphorylating); BfMnSOD: manganese superoxide dismutase.

**Gene (Primer) names**	**Primer sequences（5'→3'）**
**Race PCR**	
BfPMHA-R1 (5’)	CGTGCCAATGGCCGTCAGCGTC
BfPMHA-R2 (5’)	CCCGTGGCCACAACGACCGCAG
BfPMHA-F1 (3’)	GCGGGCGGGCATTACGGAGCT
BfPMHA-F2 (3’)	GAGCTCCGCTTTGCCCCGTTTGAT
**RT-PCR**	
BfPMHA-F	CGCGTCGTCGTCACCTTTTCG
BfPMHA-R	CGTCCGTGGAGATGGACAGCAT
Efly-F	CGAGTGGGACCAGGAGGGTAAT
Efly-R	CCCACGCAAAGTAGTCCTCAACAA
**Protein interaction analysis**	
pBT3-SUC-F	TGGCATGCATGTGCTCTG
pBT3-SUC-R	GTAAGGTGGACTCCTTCT
pPR3-N-F	GTCGAAAATTCAAGACAAGG
pPR3-N-R	AAGCGTGACATAACTAATTAC
pBT3-SUC-*BfPMHA*-F	CAAAATATCTGCAATGGCCATTACGGCCATGGCGTCGTCCACCTCGTCC
pBT3-SUC-*BfPMHA*-R	CGAATTCCTGCAGATGGCCGAGGCGGCCTCATTTCTTTCCCGGAACCCC
**BIFC**	
BfMnSOD-F	CTGGCGCGCCACTAGTGGATCCATGGCGTTTGCGCTGCCCCCCCTG
BfMnSOD-R	GTACATCCCGGGAGCGGTACCAGCCATCGGCGTGTCAAACGCGG
BfFBA-F	CTGGCGCGCCACTAGTGGATCCATGGCGTTTGTTGCTGCTGCC
BfFBA-R	GTACATCCCGGGAGCGGTACCATACTTGTACCCGGCCTCAAAGGTG
BfGAPDH-F	CTGGCGCGCCACTAGTGGATCCATGCGCTCGGTCAATGGCGC
BfGAPDH-R	GTACATCCCGGGAGCGGTACCGGCCCACTTGGAGGCCATGATC

Table S2 Supplemented information of plasma membrane H^+-ATPase in others organism.

Code	organism	accesion number
Bangia fuscopurpurea	*Bangia fuscopurpurea*	OQ363809
Lupinus albus	*Lupinus albus*	AAY42949.1
Oryza sativa Japonica Group	*Oryza sativa Japonica Group*	CAD29297.1
Populus alba	*Populus alba*	AAN87804.1
Arabidopsis thaliana	*Arabidopsis thaliana*	NP_001318282.1
Daucus carota	*Daucus carota*	BAD16684.1
Zea mays	*Zea mays*	NP_001346412.1
Nicotiana plumbaginifolia	*Nicotiana plumbaginifolia*	AAA34099.1
Solanum lycopersicum	*Solanum lycopersicum*	NP_001234775.1
Solanum tuberosum	*Solanum tuberosum*	XP_006367061.1
Homo sapiens	*Homo sapiens*	NP_001001396.1
Aspergillus fumigatus	*Aspergillus fumigatus*	AAK94755.1
Saccharomyces cerevisiae	*Saccharomyces cerevisiae*	GHM90152.1
Aureobasidium melanogenum	*Aureobasidium melanogenum*	KAH0357736.1
Saccharomyces boulardii	*Saccharomyces boulardii*	KQC40590.1
Blumeria graminis	*Blumeria graminis*	AAK94188.1
Aspergillus puulaauensis	*Aspergillus puulaauensis*	XP_041551158.1
Ceratobasidium sp.	*Ceratobasidium sp.*	KAG9088733.1
Tulasnella sp.	*Tulasnella sp.*	KAG9027398.1
Lacticaseibacillus paracasei	*Lacticaseibacillus paracasei*	WP_108298830.1
Mycobacterium marinum	*Mycobacterium marinum*	WP_117436307.1
Acidithiobacillus thiooxidans	*Acidithiobacillus thiooxidans*	WP_215891525.1
Rhodovulum sulfidophilum	*Rhodovulum sulfidophilum*	WP_202923962.1
Acidithiobacillus ferrooxidans	*Acidithiobacillus ferrooxidans*	WP_215854205.1
Pandoraea pnomenusa	*Pandoraea pnomenusa*	WP_063599650.1
Fimbriiglobus ruber	*Fimbriiglobus ruber*	OWK37340.1
Paraburkholderia kururiensis	*Paraburkholderia kururiensis*	WP_042301756.1
Acetobacter thailandicus	*Acetobacter thailandicus*	WP_212381558.1
Haplosporangium sp.	*Haplosporangium sp.*	KAF9194881.1
Mortierella polycephala	*Mortierella polycephala*	KAG0264310.1
Mortierella sp.	*Mortierella sp.*	KAG0239244.1
Lunasporangiospora selenospora	*Lunasporangiospora selenospora*	KAF9583394.1
Dissophora globulifera	*Dissophora globulifera*	KAG0307480.1
Gryganskiella cystojenkinii	*Gryganskiella cystojenkinii*	KAG0055977.1
Daedalea quercina	*Daedalea quercina*	KZT74479.1
Cortinarius glaucopus	*Cortinarius glaucopus*	KAF8812585.1
Mortierella claussenii	*Mortierella claussenii*	KAI1318967.1
Acaromyces ingoldii	*Acaromyces ingoldii*	XP_025374687.1
Actinomortierella wolfii	*Actinomortierella wolfii*	KAG0229395.1
Antrodia serialis	*Antrodia serialis*	KAH9938690.1
Modicella reniformis	*Modicella reniformis*	KAG0006558.1
Dissophora ornata	*Dissophora ornata*	KAF8932225.1
Gamsiella multidivaricata	*Gamsiella multidivaricata*	KAG0354592.1
Podila humilis	*Podila humilis*	KAG0346348.1
Mortierella alpina	*Mortierella alpina*	KAF9289521.1
Fomitopsis rosea	*Fomitopsis rosea*	KAH9843774.1
Haplosporangium bisporale	*Haplosporangium bisporale*	KAF8950897.1
Podila horticola	*Podila horticola*	KAF9320009.1
Fomitopsis betulina	*Fomitopsis betulina*	KAI0733405.1
Puccinia sorghi	*Puccinia sorghi*	KNZ45000.1
Podila minutissima	*Podila minutissima*	KAG0357995.1

Actinomortierella ambigua	*Actinomortierella ambigua*	KAF9979015.1
Podila epicladia	*Podila epicladia*	KAG0075695.1
Linnemannia zychae	*Linnemannia zychae*	KAF9927964.1
Entomortierella chlamydospora	*Entomortierella chlamydospora*	KAG0006177.1
Coprinopsis sp.	*Coprinopsis sp.*	KAH6896302.1
Mycena venus	*Mycena venus*	KAF7352277.1
Podila verticillata	*Podila verticillata*	KAF9374995.1
Podila clonocystis	*Podila clonocystis*	KAG0032371.1
Linnemannia gamsii	*Linnemannia gamsii*	KAG0312153.1
Linnemannia schmuckeri	*Linnemannia schmuckeri*	KAF9151623.1
Linnemannia hyalina	*Linnemannia hyalina*	KAG9070755.1
Mortierella hygrophila	*Mortierella hygrophila*	KAF9548418.1
Linnemanniae longata	*Linnemanniae longata*	OAQ36331.1
Puccinia graminis f. sp.	*Puccinia graminis f. sp.*	KAA1113059.1
Microcystis aeruginosa	*Microcystis aeruginosa*	WP_193956096.1
Candidatus Nitrosotalea bavarica	*Candidatus Nitrosotalea bavarica*	WP_101476794.1
Aciduliprofundum boonei	*Aciduliprofundum boonei*	WP_008085723.1
Candidatus Nitrosotalea okcheonensis	*Candidatus Nitrosotalea okcheonensis*	WP_157927216.1
Acidiplasma	*Acidiplasma*	WP_048102021.1
Picrophilus torridus	*Picrophilus torridus*	WP_011178283.1
Picrophilus oshimae	*Picrophilus oshimae*	WP_236719373.1
Cyanidium caldarium	*Cyanidium caldarium*	BAA20486.1
Porphyridium purpureum	*Porphyridium purpureum*	KAA8496232.1
Dunaliella acidophila	*Dunaliella acidophila*	AAB49042.1
Coccomyxa subellipsoidea	*Coccomyxa subellipsoidea*	XP_005647875.1
Chlamydomonas reinhardtii	*Chlamydomonas reinhardtii*	CAC19368.1
Klebsormidium nitens	*Klebsormidium nitens*	GAQ79028.1
Pycnococcus provasolii	*Pycnococcus provasolii*	GHP11804.1
Ulva lactuca	*Ulva lactuca*	UFQ04581.1
Scenedesmus sp.	*Scenedesmus sp.*	KAF6256632.1
Phytophthora infestans	*Phytophthora infestans*	AAQ23136.1
Aphanomyces cochlioides	*Aphanomyces cochlioides*	KAG9399895.1
Achlya hypogyna	*Achlya hypogyna*	OQR93764.1
Phytophthora nicotianae	*Phytophthora nicotianae*	ABF85691.1
Trypanosoma cruzi	*Trypanosoma cruzi*	KAF5219432.1
Leishmania major strain Friedlin	*Leishmania major strain Friedlin*	XP_001682561.1
Bodo saltans	*Bodo saltans*	CUF89536.1
Trypanosoma brucei gambiense	*Trypanosoma brucei gambiense*	XP_011778672.1
P-type *Dunaliella acidophila*	*Dunaliella acidophila*	AAB49042.1
V-type *Ectocarpus siliculosus*	*Ectocarpus siliculosus*	CBJ48318.1
F-type *Ectocarpus siliculosus*	*Ectocarpus siliculosus]*	CBJ32298.1
V-type *Porphyra tenera*	*Porphyra tenera*	JC7151
V-type *Porphyridium purpureum*	Porphyridium purpureum	KAA8496232.1
P-type *Phaeodactylum tricornutum*	*Phaeodactylum tricornutum*	XP_002185425.1
P-type *Chlamydomonas reinhardtii*	Chlamydomonas reinhardtii	CAC19368.1
P-type *Ulva lactuca*	Ulva lactuca	UFQ04581.1

Table S3 Positive clones that encoding different proteins.

Number of Clones	NCBI Accession	NCBI Description
1	XM_037714372.1	PREDICTED: *Dermacentor silvarum* 60S ribosomal protein L27a-like, mRNA
2	XM_015798350.1	*Leptomonas pyrrhocoris* 40S ribosomal protein S14 mRNA
4	XM_030473944.1	PREDICTED: *Strigops habroptila* V-type proton ATPase catalytic subunit A-like, mRNA
7、9	JX416800.1	*Porphyra haitanensis* isolate Z-61 manganese superoxide dismutase mRNA, complete cds
8	AB127048.1	*Porphyra yezoensis* mRNA for fructose-bisphosphate aldolase, complete cds
10	DQ914563.1	*Porphyra yezoensis* polyubiquitin (PUBI-1) gene, complete cds
13	XM_031267007.1	PREDICTED: *Ipomoea triloba* calmodulin-7, mRNA
15	AB127051.1	*Porphyra yezoensis* mRNA for glyceraldehyde 3-phosphate dehydrogenase ($NADP^+$) (phosphorylating), complete cds
23	LR597439.1	*Salarias fasciatus* genome assembly, chromosome: 4
27	CP051059.1	*Aspergillus flavus* strain A1 chromosome 1
28	JN991055.1	*Porphyra haitanensis* strain Z-61 40S ribosomal protein S15a mRNA, complete cds
30	XM_005709990.1	*Chondrus crispus* unnamed protein product mRNA, complete cds

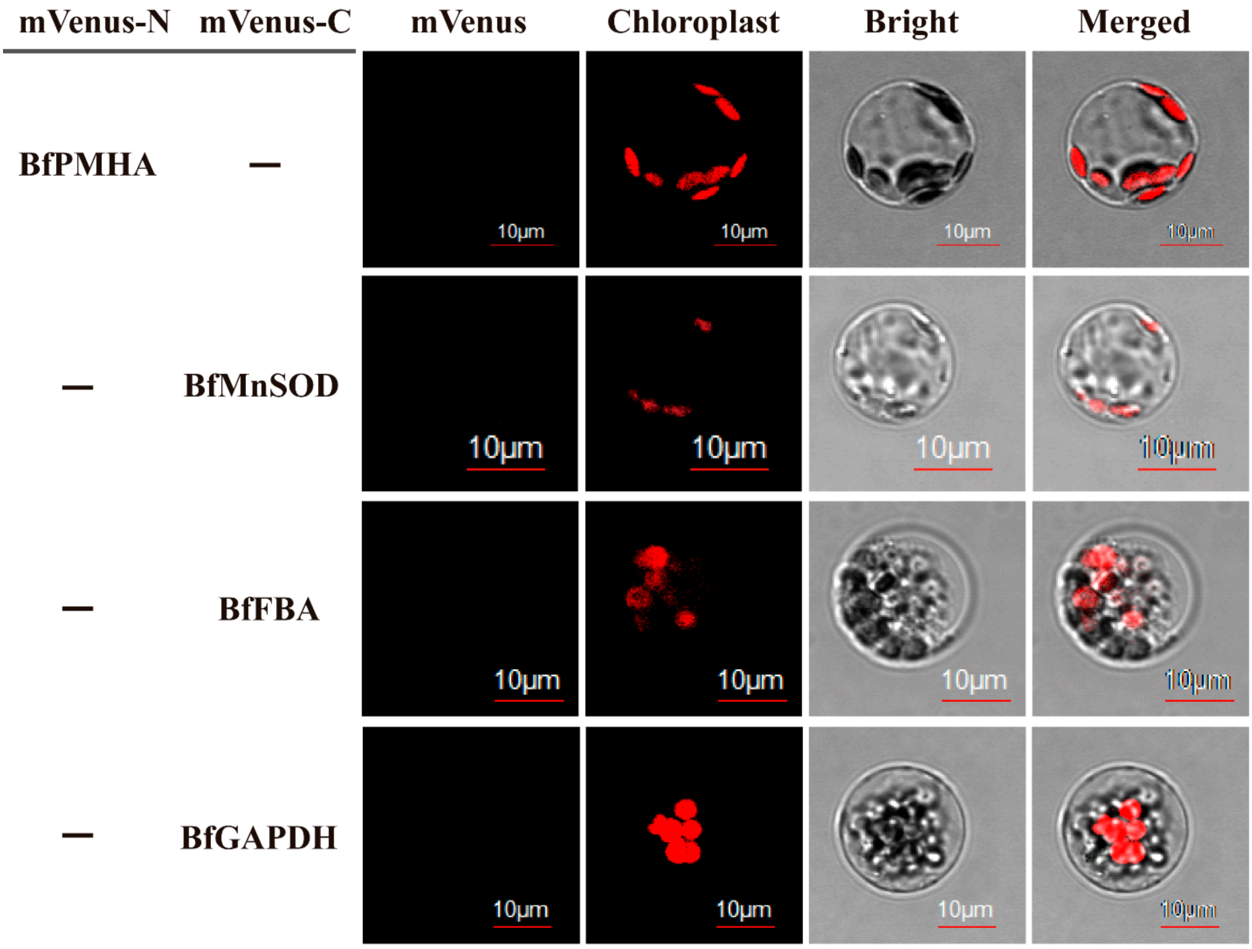

Figure S1. The negative control for BfPMHA interact with BfMnSOD, BfFBA and BfGAPDH, separately, in rice protoplasts by BiFC assays. mVenus, yellow fluorescence. Chloroplast, red fluorescence. Scale bar, 10 μm.
